# Classification of cervical vertebral maturation stages with machine learning models: leveraging datasets with high inter- and intra-observer agreement

**DOI:** 10.1186/s40510-024-00535-1

**Published:** 2024-09-16

**Authors:** Potjanee Kanchanapiboon, Pitipat Tunksook, Prinya Tunksook, Panrasee Ritthipravat, Supatchai Boonpratham, Yodhathai Satravaha, Chaiyapol Chaweewannakorn, Supakit Peanchitlertkajorn

**Affiliations:** 1 Division of Nuclear Medicine, Department of Radiology, Faculty of Medicine Siriraj Hospital, Mahidol University, 2 Wang Lang Rd, Siriraj, Bangkok Noi, Bangkok, 10700 Thailand; 2 Department of Orthodontics, Faculty of Dentistry, Mahidol University, 6 Yothi Rd, Thung Phaya Thai, Ratchathewi, Bangkok, 10400 Thailand; 3 Private Practice, Bangkok, Thailand; 4 Department of Biomedical Engineering, Faculty of Engineering, Mahidol University, 999 Phutthamonthon 4 Rd, Salaya, Nakhon Pathom 73170 Thailand

**Keywords:** Cervical vertebral maturation stages, Machine learning, Artificial intelligence, Consensus-based model, Landmark annotation

## Abstract

**Objectives:**

This study aimed to assess the accuracy of machine learning (ML) models with feature selection technique in classifying cervical vertebral maturation stages (CVMS). Consensus-based datasets were used for models training and evaluation for their model generalization capabilities on unseen datasets.

**Methods:**

Three clinicians independently rated CVMS on 1380 lateral cephalograms, resulting in the creation of five datasets: two consensus-based datasets (Complete Agreement and Majority Voting), and three datasets based on a single rater’s evaluations. Additionally, landmarks annotation of the second to fourth cervical vertebrae and patients’ information underwent a feature selection process. These datasets were used to train various ML models and identify the top-performing model for each dataset. These models were subsequently tested on their generalization capabilities.

**Results:**

Features that considered significant in the consensus-based datasets were consistent with a CVMS guideline. The Support Vector Machine model on the Complete Agreement dataset achieved the highest accuracy (77.4%), followed by the Multi-Layer Perceptron model on the Majority Voting dataset (69.6%). Models from individual ratings showed lower accuracies (60.4–67.9%). The consensus-based training models also exhibited lower coefficient of variation (CV), indicating superior generalization capability compared to models from single raters.

**Conclusion:**

ML models trained on consensus-based datasets for CVMS classification exhibited the highest accuracy, with significant features consistent with the original CVMS guidelines. These models also showed robust generalization capabilities, underscoring the importance of dataset quality.

## Background

Determining the optimal age for orthodontic treatment has been a topic of considerable debate. Favourable treatment timing is critical in achieving desirable treatment outcomes and efficiency [1]. Starting treatment either too early or too late can prolong care or complicate processes [2, 3]. Orthodontists traditionally determine treatment timing by assessing hand-wrist radiographs [4]. The British Orthodontic Society currently discourages this method for due to concerns over additional radiation exposure [5]. Instead, several studies advocated using cervical vertebral maturation stage (CVMS) assessed on a lateral cephalogram, a standard radiographic record for orthodontic diagnosis and treatment planning [6–13]. CVMS was found to correlate well with hand-wrist maturity, suggesting that CVMS could serve as an alternative for assessing skeletal maturity [14]. Baccetti et al. [12] proposed a CMVS guideline that is widely adopted in research and clinical practice. They described six cervical stages (CS) as follows: CS-1 and 2 mark a period preceding the peak mandibular growth, the mandibular growth peak is observed between CS-3 and 4, CS-5 represents a post-peak phase, and CS-6 indicates the end of mandibular growth [12]. Manual CVMS interpretation relies on subjective assessments. This resulted in inconsistency and inaccuracy according to previously published studies demonstrating low to moderate intra- and inter-observer reliability [15, 16].

There is a growing interest in employing artificial intelligence (AI) in orthodontics for automating tasks such as orthodontic diagnoses and treatment planning [17], determining the need for extractions [18], orthodontic model analysis [19], and CVMS classification [20–31]. Machine learning (ML) and deep learning (DL) are subsets of AI techniques. ML focuses on training a machine to perform a specific task with structured and labeled data. DL targets complex tasks with unstructured data using artificial neural networks to emulate the human brain’s learning process [32]. ML models were commonly used for CVMS classification in the beginning [20–25]. Recently, DL models have been increasingly utilized for this task [26–31]. Despite their growing popularity, the complexity of DL models, and challenges in understanding their multi-layered neural networks pose difficulties in fully comprehending the basis of their decisions-making process [33]. The primary focus of past studies, whether utilizing DL or ML models, was directed towards assessing the accuracy of the models [20–31]. However, it is equally important to consider other factors such as the reliability and consistency of the models’ predictions. An AI model may perform well under certain conditions but could fail to generalize across unseen datasets [34]. Although it is critical to ensure that AI models are trained on accurate and unbiased data, most previously published studies employed only a single or two raters to classify CVMS for the purpose of training AI models [20–23, 26–30]. A reliance on the judgement of a single rater as he/she could introduce individual bias and potentially misrepresent the true CVMS classifications, and ultimately affect the overall reliability and generalizability of the models [34].

In AI, “features” refer to distinct characteristics or attributes of an image or other type of data that AI models can use to make predictions or classifications [35]. For example, features in lateral cephalogram analysis, may include angulations or distances measured between landmarks. Hence, “feature selection” plays a crucial role in ML by identifying key variables in a dataset that significantly impact the decision making process of models, thereby increasing ML models’ precision [36]. This technique is particularly relevant for improving the accuracy of CVMS classification using ML.

The effectiveness of AI models depends largely on the accuracy of their outcomes which varies according to the quality of input data, the consistency of data standards, and observer agreements [37]. Therefore, the primary objectives of this study are to assess the accuracy of ML models in classifying CVMS when applying a consensus-based method employing a panel of raters and a feature selection to the methodology, and to examine these models’ ability to generalize to unseen datasets.

## Methods

**Fig. 1:**
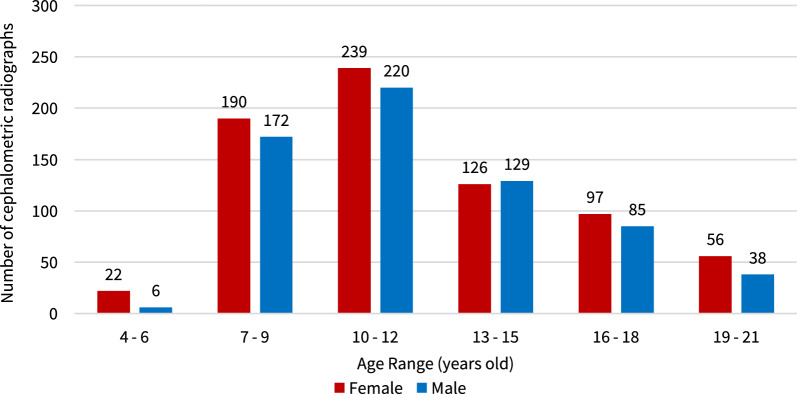
The sample distribution by gender and age

The study protocol was approved by the Mahidol University Institutional Review Board, Faculty of Dentistry/Pharmacy, with the approval number CoA No. MU-DT/PY-IRB 2022/015.2803. Data were collected from lateral cephalograms taken as part of routine orthodontic records at the Department of Orthodontics, Faculty of Dentistry, Mahidol University. The radiographic images were captured with KODAK 9000C device (Eastman Kodak Company, Rochester, NY, USA) with exposure settings of 80 kVp, 8 mA, and 1 s. For sample size determination, we employed a heuristic approach, using large and well balanced datasets to ensure robust training and validation of models [38]. The samples for this study comprised 1380 lateral cephalograms from individuals aged between 4 and 21 years. The female to male ratio was 1.12:1. The sample distribution by gender and age was presented in Fig. 1.

### Inclusion criteria

- Lateral cephalograms taken in a natural head position.
- Lateral cephalograms of adequate quality that clearly show the second to fourth cervical vertebrae (C2–C4).

### Exclusion criteria

- Lateral cephalograms that are not of standard quality such as blurry or noisy images.

#### CVMS classification by a panel of raters

The CVMS classification in this study was performed following the method described by Baccetti et al. [12]. All cephalograms were independently classified by a panel of raters (one experienced orthodontist in academia, one experienced orthodontist in private practice, and one orthodontic resident). The first two raters have 20 years of experience in orthodontics, while the last one is a senior orthodontic resident in a program where CVMS classification is routinely utilized as a part of diagnosis and treatment planning. A calibration session was conducted to reduce personal bias and increase inter-observer reliability prior to individual CVMS rating. Each rater then independently evaluated the CVMS on all cephalograms. After one-month interval, they repeated the process on a set of 35 randomly selected radiographs. Intra- and inter-observer agreements for the CVMS rating were calculated using Weighted Kappa statistics.

#### Dataset preparation

The dataset from three raters underwent a data preparation process that employed a consensus-based approach, using Python software, Version 3.9.7 (Python Software Foundation, Fredericksburg, VA, USA). This approach grouped CVMS assessments into “Complete Agreement” (all raters provided the same rating), and “Partial Agreement” (two out of three agreed on the rating). Finally, five datasets were created for model training: three individual datasets from each of the three raters (termed Rater 1, Rater 2, and Rater 3 datasets), two consensus-based datasets: “Complete Agreement”, and a “Majority Voting” (a combination of “Complete Agreement” and “Partial Agreement”). Cephalograms which all three raters provided differing CVMS ratings (a complete disagreement) were excluded.

#### Landmarks annotation

**Fig. 2:**
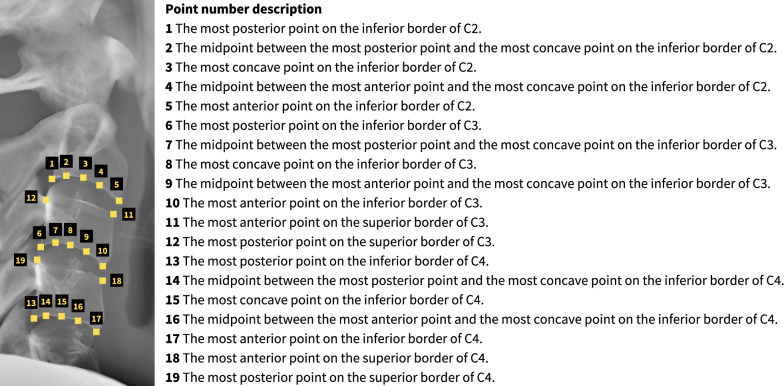
Definitions of landmarks on lateral cephalogram

An additional stage of data extraction for this study was performed by annotating landmarks around the cervical bones on lateral cephalograms. We utilized VGG Image Annotator software, Version 2.0.10 (Department of Engineering Science, University of Oxford, Oxford, UK) to identify 19 landmarks surrounding the C2–C4, and created various features with those landmarks. The definition of each point is detailed in Fig. 2. The pixel coordinates of all points were subsequently exported and processed using the Python software to extract C2, C3 and C4 features.

#### Feature selection

Feature selection involves identifying and retaining only the most impactful variables for model training. This process enhances accuracy and efficiency, while reducing overfitting and computational costs [39]. We accomplished this by utilizing a Random Forest model, which created several decision trees to make more reliable and accurate predictions. The features input into each dataset were classified into four groups: the general information feature group (patient’s age and gender), and the C2, C3, and C4 feature groups. The last three groups consisted of measurements such as distance, angles, and area calculated from the annotated landmarks on the C2–C4.

Data in each of the five datasets were then randomly divided into a training set (70%) and a testing set (30%). The prediction pipeline for each model is built using the Python software which serves as the main programming language, together with two additional tools: the scikit-learn, Version 1.0.2 and scikit-optimize libraries, Version 0.9.0 (Python Software Foundation, Fredericksburg, VA, USA) [40].

#### CVMS classification by ML models

This phase determined the model that exhibited the highest accuracy for each dataset, referred to as top-performing models. This was accomplished through hyperparameter tuning, a process which determines optimal parameters for each model to make accurate predictions on a given dataset [41]. Only relevant features, identified in the feature selection step, were input into the six ML models including Logistic Regression (LogReg), Multi-Layer Perceptron (MLP), Random Forest (RForest), K-Neighbors, Support Vector Machine (SVM), and Gradient Boosting (GraBoost).

#### Model generalization

**Fig. 3:**
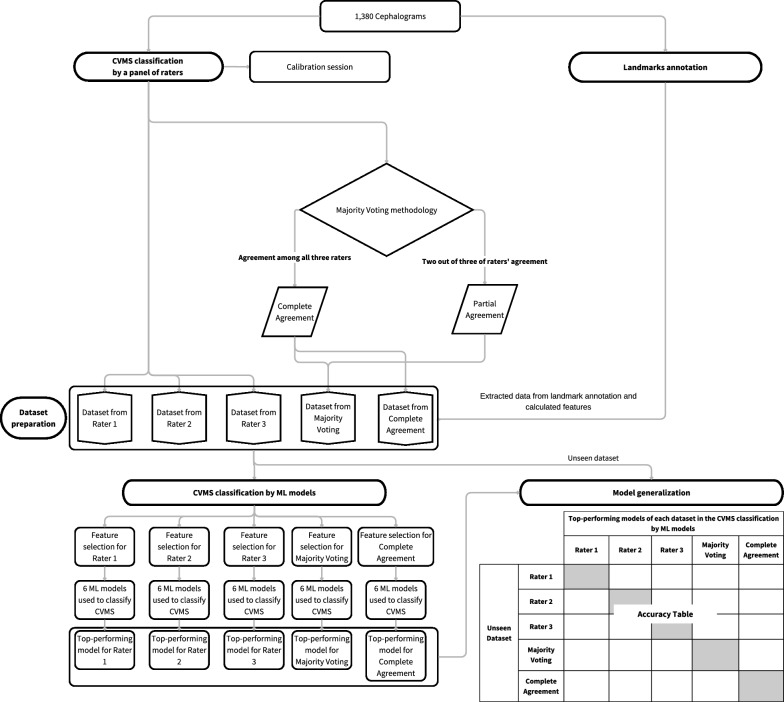
Flowchart of the methodological approach in this study. A panel of three raters attended a calibration session before rating the CVMS independently. Subsequently, five datasets including two from the consensus-based approach, were created from the ratings. Landmarks annotation of second to fourth cervical vertebrae on lateral cephalograms was also performed. These datasets then underwent feature selection and CVMS classification using ML models. The outcome of this phase is the accuracy of ML models for each dataset. Finally, the five top-performing models were deployed to evaluate their accuracy in predicting CVMS on four other unseen datasets

To evaluate model generalization and ensure its robust performance on new, unseen data, two critical steps were taken. First, 30% of cephalograms for each stage of CVMS in each dataset were randomly selected, ensuring the original data distribution was maintained. This process aimed to create a balanced test set that accurately reflected a variety of cases the models might encounter in real-world applications. Next, top-performing models from all five datasets identified in the previous phase (CVMS classification by ML models), were applied to four other unseen datasets (five original datasets minus the dataset from which each model originated). This cross-dataset evaluation enabled the assessment of each model’s ability to make accurate predictions and effectively generalize across different data sets. This demonstrated their potential applicability and reliability in broader clinical settings. The overview of this study methodology is depicted in Fig. 3.

#### Statistical analysis

The model’s performance was evaluated using classification accuracy, based on the data in the testing set. Mean, standard deviation (SD), and coefficient of variation (CV) were employed to assess the model’s generalization capability and facilitate comparative analysis of variability across datasets. CV, representing the ratio of the standard deviation to the mean, provides a standardized measure of variability that can be compared across different datasets. A lower CV indicates less variability relative to the mean, suggesting greater consistency and reliability within the dataset and vice versa [42]. All statistical calculations were performed using the Python software.

## Results

### Intra- and inter-observer reliability

Intra-observer agreement demonstrated strong agreement, with values ranging from κ = 0.86 to 0.92. Inter-observer agreement values ranged from κ = 0.62 to 0.78, indicating moderate agreement (P < 0.05) [43] (Table 1).

**Table 1:** Intra- and inter-observer agreement

	Rater	Weighted kappa	95% CI	SD
Intra-observer agreement	1	0.86	0.78–0.95	0.04
	2	0.90	0.82–0.98	0.04
	3	0.92	0.85–0.98	0.03
Inter-observer agreement	1 versus 2	0.78	0.76–0.8	0.01
	1 versus 3	0.62	0.59–0.64	0.01
	2 versus 3	0.68	0.67–0.70	0.01

CI, confidence interval; SD, standard deviation

### Sample size of each dataset

There were 456 (33.04%) subjects identified as Complete Agreement, and 812 (58.84%) as Partial Agreement. Therefore, 1268 (91.88%) radiographs fell within the Majority Voting category. Only 112 (8.12%) radiographs received complete disagreement from all raters. Approximately 30% of each dataset, a total of 414 radiographs, were randomly selected to evaluate model generalization. Of these, 137 (33.09%) radiographs demonstrated Complete Agreement, and 244 (58.93%) as Partial Agreement. As a result, the Majority Voting category comprised a sample size of 381 (92.03%) radiographs. The sizes and distribution of each dataset in both stages are presented in Table 2.

**Table 2:** Sample distribution in two phases: “CVMS classification by ML models” and “Model generalization”

	CVMS classification by ML models					Model generalization				
	Rater 1	Rater 2	Rater 3	Majority Voting	Complete Agreement	Rater 1	Rater 2	Rater 3	Majority Voting	Complete Agreement
CS-1	201	182	204	181	98	60	56	63	58	31
CS-2	151	215	261	187	60	45	69	83	53	19
CS-3	181	277	210	188	41	59	77	59	51	9
CS-4	328	237	353	256	129	95	72	101	73	42
CS-5	294	218	294	255	79	87	60	90	83	19
CS-6	225	251	58	201	49	68	80	18	63	17
Total	1380	1380	1380	1268	456	414	414	414	381	137

### Feature selection

**Fig. 4:**
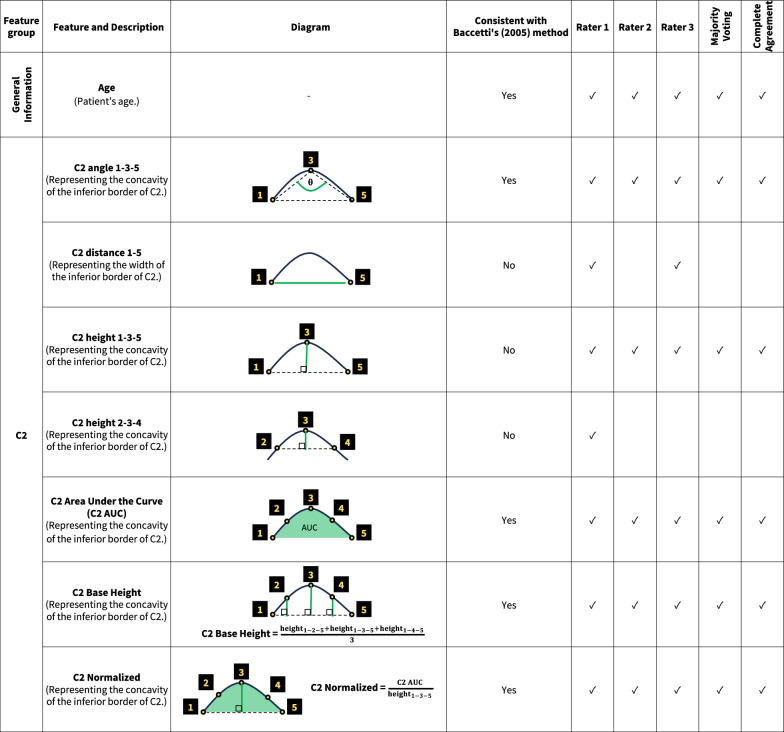

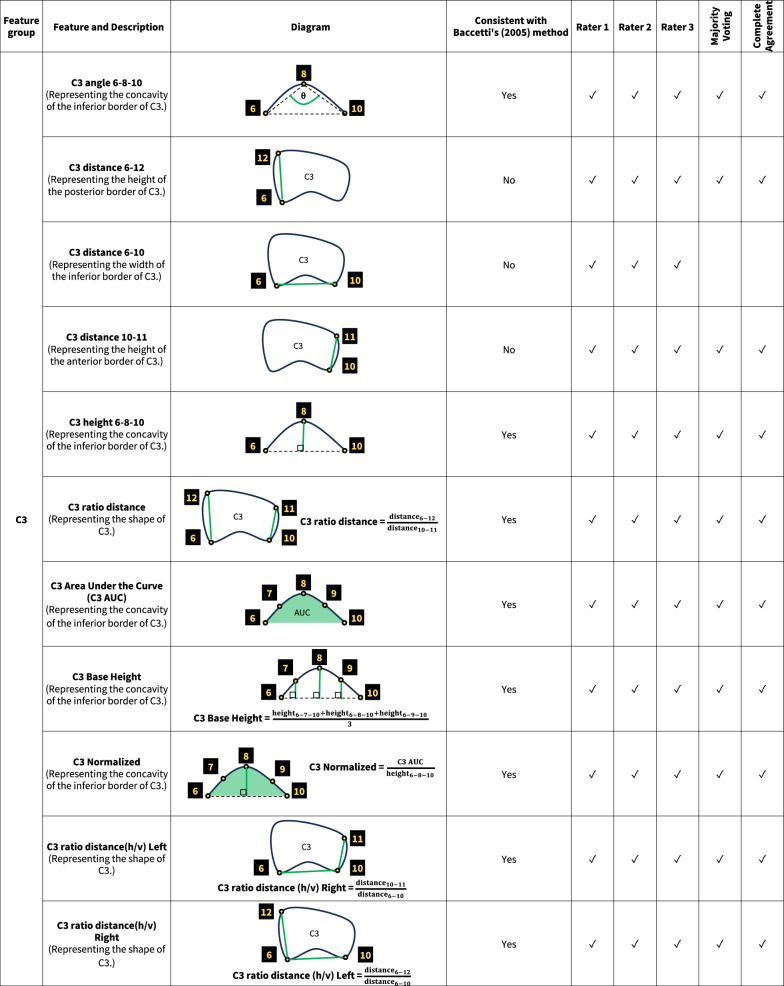

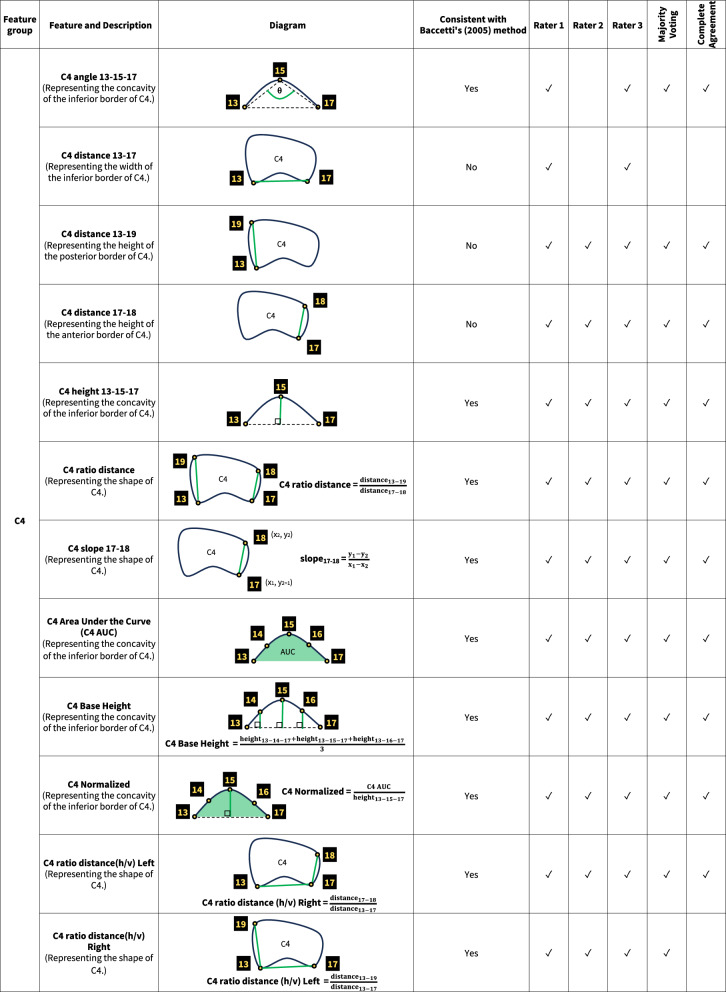
Significant features on C2–C4 for CVMS classification according to features selection

The feature selection process identified a total of 31 features as significant across five datasets. (Fig. 4) Some features were considered significant in all five datasets, while others were specific to certain datasets. Within the general information feature group, the feature “Age,” (patient’s age) was consistently selected as significant across all datasets. This underscored the importance of patient age over gender in influencing ML model outcomes for CVMS classification.

In the C2 feature group, “C2 angle 1–3–5” and “C2 height 1–3–5” were significant features which illustrated C2’s concavity, a key feature according to Baccetti et al. [12]. Their significance across all datasets underlined the concavity at the inferior border of C2 as a crucial criterion for accurate CVMS staging. Evaluating the concavity at the inferior border of C3 and C4 was also essential. Features such as “angle,” “height,” and “area under curve (AUC)” were neccessary for assessing the concavity. And they were considered significant across all datasets. In addition, the analysis of C3 and C4 took into consideration of the vertebral shapes (trapezoidal, horizontally rectangular, square, or vertically rectangular). In this study, features denoted by “ratio” represented the shape of these bones. All “ratio” features were deemed significant across datasets except “C4 ratio distance(h/v) Right”. Some features were considered significant in individual rater datasets but not consistent with Baccetti et al. [12]. For example, “C3 distance 6–10” (the width of C3’s inferior border) was identified as significant in three individual rater datasets but was insignificant in the Complete Agreement and Majority Voting datasets.

### CVMS classification by ML models

**Fig. 5:**
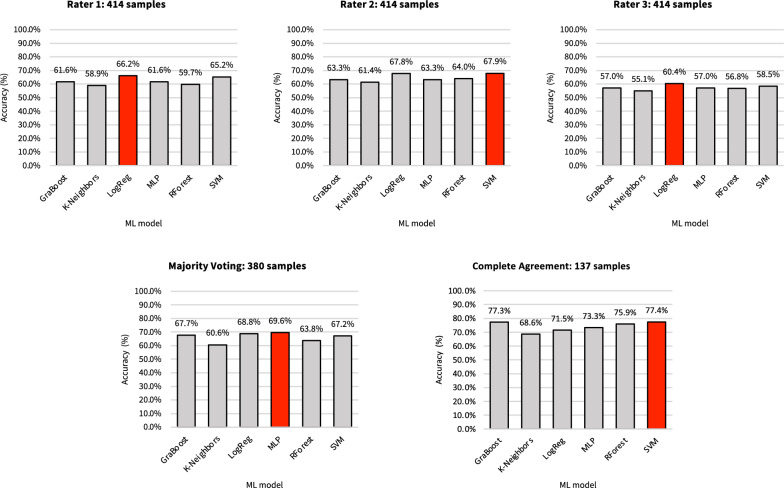
Classification accuracy of the trained models on CVMS classification in five datasets

Among the five datasets, the Complete Agreement dataset exhibited the highest accuracy of 77.4% with the Support Vector Machine (SVM) model. The Majority Voting dataset had the second highest accuracy of 69.6% utilizing the Multi-Layer Perceptron (MLP) model. For the single rater datasets, Rater 2 obtained the highest accuracy at 67.9% with the SVM model. Rater 1 achieved an accuracy of 66.2% using the MLP model. And Rater 3 attained an accuracy of 60.4% with Logistic Regression (LogReg) model. The accuracy of all trained models was presented in Fig. 5

### Model generalization

Top-performing models from each dataset were tested on four other unseen datasets to assess their generalization. Their accuracies are displayed in Table 3. Top-performing model by Rater 2, achieved the highest mean accuracy of 62.5%, followed by the Majority Voting model at 61.8%. The remaining models had accuracies of less than 60%. Despite achieving a mean accuracy of 57.6%, the Complete Agreement model demonstrated the lowest standard variation (0.03). Furthermore, the Majority Voting model also exhibited less variation than those of single raters, indicating a reduction in subjective interpretation. For generalization across unseen datasets, the models were ranked based on increasing CV values as follows: Complete Agreement, Majority Voting model, and models from single raters.

**Table 3:** The assessment of model generalization in CVMS classification across all datasets

		Top-performing models of each dataset in the CVMS classification by ML models				
		Rater 1	Rater 2	Rater 3	Majority Voting	Complete Agreement
New and unseen dataset	Rater 1	x	60.5%	42.6%	61.0%	55.2%
	Rater 2	59.1%	x	49.9%	62.5%	55.7%
	Rater 3	45.0%	46.5%	x	49.2%	57.4%
	Majority Voting	63.6%	69.4%	54.9%	x	62.3%
	Complete Agreement	68.6%	73.7%	65.7%	74.5%	x
Average accuracy		59.1%	62.5%	53.3%	61.8%	57.6%
SD		0.10	0.12	0.10	0.10	0.03
CV		0.17	0.19	0.18	0.17	0.06

Top-performing models from each dataset (columns) were tested on four new, unseen datasets (rows). Generalizability was determined by coefficient of variation (CV) and standard deviation (SD)

## Discussion

This study demonstrated that applications of ML models in CVMS classification utilizing datasets with high inter- and intra-observer agreement improved diagnostic accuracy and reliability. This approach reduced subjective bias associated with individual assessments. This study also incorporated feature selection into its methodology. The results found that age and features related to C2–C4’s morphology were significant and consistent with the description by Baccetti et al. [12]. Additionally, model generalization showed that the consensus-based approach resulted in a better performance in terms of accuracy and reliability than single raters on unseen datasets.

Santiago et al. reported the CVMS’s poor reliability and validity, suggesting the difficulty of consistent and accurate assessments [44]. However, our study achieved higher intra- and inter-observer agreement in CVMS classification (κ = 0.86 to 0.92 and κ = 0.62 to 0.78) than previously reported low to moderate levels of agreements [15, 16]. Our results also exceeded the substantial agreement levels noted by Rainey et al. (κ = 0.6 to 0.8, inter-observer κ = 0.68) [45]. This improvement could be attributed to the calibration session, which minimized discrepancies and variations in the assessment process, leading to greater agreement among observers. The less than perfect inter-observer agreement reflects the inherent variability of opinions among raters [46]. This variability was expected due to differences in raters’ experience and the subjective nature of visual assessments [47]. In fact, this supports the utility of AI in clinical orthodontics, where obtaining a consensus among orthodontists is not always possible.

Prior studies often relied on a single rater to train AI models [20–23, 26, 27] to simplify the process, but could potentially introduce bias. The variability in individual interpretations [15, 16] raises questions about the effectiveness of models trained solely on such data. Mathew et al. highlighted in a systematic review that diagnostic accuracy fluctuates due to variations in the quality of input data and a lack of standardization including intra- and inter-observer agreement [34]. Our methodology mitigated the issue of relying on a single rater for AI training due to CVMS classification’s inherent subjectivity by utilizing a panel of raters. The inclusion of patient’s age, and C2–C4’s morphology further enhanced the accuracy of classifications.

While a few studies involved two raters to improve reliability [28–30], our study employed a panel of three raters. Moreover, our study utilized a consensus-based mechanism to create datasets for models training. We believe that it is an innovative method that reduced subjectivity and bias. This marks our research as the first to apply the approach specifically to this task. It emphasized the importance of a consensus among raters in refining AI model training for improved diagnostic accuracy.

The Majority Voting dataset had a sample size of 1268 cephalograms, surpassing the typical range of 236 to 1018 samples reported in previous studies [20–25, 27–30]. This is a high quality dataset not only in terms of sample size but also in balance across different datasets. On the contrary, the scarcity of the Complete Agreement dataset highlighted the difficulty in obtaining unanimous consensus among all raters and underscored the challenges in curating datasets of this nature. It also reflects the preparation required to attain a high level of reliability. Despite its smaller sample size (456 samples), the Complete Agreement dataset achieved the highest accuracy (77.4%) in our study. These results suggested that when all raters are in complete agreement, the data quality increased as demonstrated by the better accuracy achieved. Santiago et al. [20] obtained a high accuracy rate of 81.4% but used a relatively small dataset consisting of only 236 samples. Such high accuracy in a small dataset may predispose to a potential risk of overfitting. Overfitting occurs when a model learns to perform exceptionally well on the specific data provided, but might not generalize effectively to unseen data. Conversely, Kim et al. [28] utilized a larger dataset comprising 720 samples but achieved a lower accuracy of 62.5%. The lower accuracy could be attributed to the increased complexity and diversity of a larger dataset which might require a more robust and generalized model. Hence, it is essential to strike a balance between dataset size and model performance when aiming to achieve both generalizability and accuracy.

To the best of our knowledge, this study is the first to incorporate feature selection into its methodology to classify CVMS with AI. The results found features related to C2–C4’s morphology significant and consistent with the description by Baccetti et al. [12]. However, features unrelated to the description by Baccetti et al. [12] such as the base width of C3 and C4 were deemed significant in individual rater datasets but not in Majority Voting nor Complete Agreement datasets. This observation further supported the advantage of employing a panel of raters over a single rater as these unrelated features might be used by individuals but were excluded by the consensus process. Another noteworthy feature in our model was the patient’s chronological age. This is very practical for everyday clinical practice since age and gender are often factors in evaluating growth and development. Age in particular can be helpful in differentiating between closely related stages. The selection of age as a significant feature substantiated the potential of employing feature selection to enhance precision in CVMS classification. It also supported a recommendation that CVMS assessment should not be performed in isolation [46].

This study is also the first to assess model generalization. Even though Rater 2’s top-performing model had the highest average performance, our analysis went beyond that. We also evaluated overall consistency and reliability across multiple datasets. The consensus-based approach’s (Complete Agreement and Majority Voting) top-performing models demonstrated more consistent results, as evidenced by a lower SD and CV across all unseen datasets. This superior consistency and reliability resulted in better generalizability for the consensus-based approach.

Many aspects in orthodontics sometimes elicit diverse opinions without a clear right or wrong answer [48]. CVMS is one such example [46]. To address this inherent variability in opinions, our study employed a consensus-based approach for CVMS classification. This approach aimed to enhance the reliability and consistency of assessments by incorporating collective expertise. Looking forward, the consensus-based methodology holds promise for application in more complex tasks, such as treatment planning, decision to extract, or decision to perform orthognathic surgery by leveraging generative AI technologies, artificial intelligence systems designed to create new content by learning patterns from existing data and producing outputs that mimic human creativity and innovation [49]. This study serves as a foundational step towards integrating AI-driven consensus methods into broader orthodontic applications, potentially improving decision-making processes in clinical practice.

Our findings highlighted the advantages of the consensus-based method with a panel of raters. This pioneering approach enhanced the reliability and accuracy of CVMS classification. ML models trained with this approach could significantly enhance their diagnostic confidence. This further supported the utility of AI in clinical orthodontics where obtaining a consensus among orthodontists is not always practical. Model generalization assessment also demonstrated that our approach yielded better consistency and reliability compared to evaluations by single raters, particularly in new and unseen cases. This suggests that our method is not only robust but also adaptable to real-world patient scenarios, making it a valuable tool for clinicians to enhance clinical decision-making and ultimately improving treatment outcomes.

### Limitations

Limitations of our study include the specificity of our sample group. The samples comprised only patients of Asian descent from a single institution. This could limit the applicability of our results to other racial groups. This concern is supported by findings from Montasser et al. which reported racial variations of the mean ages at different CVM stages [50]. Additionally, one-third of the samples consisted of children aged between 10 and 12 years. Less than ten percent was in the extreme age range groups (2% aged 4–6 years, and 7% aged 19–21 years). Therefore, future studies should include samples from various racial groups, ethnicities, and ages.

## Conclusion

In our study, ML model accuracy for CVMS classification varied among datasets. The highest accuracy was observed in the Complete Agreement dataset, followed by the Majority Voting dataset. The use of a consensus-based approach enhanced the reliability of datasets for training ML models. Feature selection confirmed that the significant features were consistent with the theoretical basis of CVMS classification by Baccetti et al. [12], especially in consensus-based datasets. The models’ successes in predicting CVMS in unseen datasets demonstrated their robust generalization capability and potential for clinical assessment.

## Data Availability

The datasets generated and/or analyzed during the current study are available in the GitHub repository named "CVMS_Classification" at the following persistent web link: https://github.com/BPK-Benz/CVMS_Classification.

## Abbreviations

CVMS: Cervical vertebral maturation stages
CS: Cervical stages
AI: Artificial intelligence
ML: Machine learning
DL: Deep learning
C2–C4: Second to fourth cervical vertebrae
LogReg: Logistic regression
MLP: Multi-layer perceptron
RForest: Random forest
SVM: Support vector machine
GraBoost: Gradient boosting
SD: Standard deviation
CV: Coefficient of variation
AUC: Area under curve

## References

[1] Proffit WR. The timing of early treatment: an overview. Am J Orthod Dentofac Orthop. 2006;129(4 Suppl):S47–9. 10.1016/j.ajodo.2005.09.014 16644417

[2] Hsieh TJ, Pinskaya Y, Roberts WE. Assessment of orthodontic treatment outcomes: early treatment versus late treatment. Angle Orthod. 2005;75(2):162–70. 15825777 10.1043/0003-3219(2005)075<0158:AOOTOE>2.0.CO;2

[3] Fleming PS. Timing orthodontic treatment: early or late? Aust Dent J. 2017;62(Suppl 1):11–9. 28297091 10.1111/adj.12474

[4] Fishman LS. Radiographic evaluation of skeletal maturation: a clinically oriented method based on hand-wrist films. Angle Orthod. 1982;52(2):88–112. 6980608 10.1043/0003-3219(1982)052<0088:REOSM>2.0.CO;2

[5] Isaacson KG, Isaacson KG, British Orthodontic S. Guidelines for the use of radiographs in clinical orthodontics. 4th ed. London: British Orthodontic Society; 2015. p. 28.

[6] Dg L. Skeletal age assessment utilizing cervical vertebrae. Pittsburgh: The University of Pittsburgh; 1972.

[7] Hassel B, Farman AG. Skeletal maturation evaluation using cervical vertebrae. Am J Orthod Dentofac Orthop. 1995;107(1):58–66. 10.1016/s0889-5406(95)70157-5 7817962

[8] Pancherz H, Szyska M. Analyse der Halswirbelkörper statt der Handknochen zur Bestimmung der skelettalen und somatischen Reife—Eine Reliabilitäts- und Validitätsuntersuchung. Inf Orthod Kieferorthop. 2000;32.

[9] O’Reilly MT, Yanniello GJ. Mandibular growth changes and maturation of cervical vertebrae–a longitudinal cephalometric study. Angle Orthod. 1988;58(2):179–84. 3164596 10.1043/0003-3219(1988)058<0179:MGCAMO>2.0.CO;2

[10] Franchi L, Baccetti T, McNamara JA Jr. Mandibular growth as related to cervical vertebral maturation and body height. Am J Orthod Dentofac Orthop. 2000;118(3):335–40. 10.1067/mod.2000.107009 10982936

[11] Baccetti T, Franchi L, McNamara JA Jr. An improved version of the cervical vertebral maturation (CVM) method for the assessment of mandibular growth. Angle Orthod. 2002;72(4):316–23. 12169031 10.1043/0003-3219(2002)072<0316:AIVOTC>2.0.CO;2

[12] Baccetti T, Franchi L, McNamara JA. The cervical vertebral maturation (CVM) method for the assessment of optimal treatment timing in dentofacial orthopedics. Semin Orthod. 2005;11(3):119–29.

[13] McNamara JA Jr, Franchi L. The cervical vertebral maturation method: a user’s guide. Angle Orthod. 2018;88(2):133–43. 29337631 10.2319/111517-787.1 PMC8312535

[14] Wong RW, Alkhal HA, Rabie AB. Use of cervical vertebral maturation to determine skeletal age. Am J Orthod Dentofac Orthop. 2009;136(4):484.e1-6 (**discussion-5**). 10.1016/j.ajodo.2007.08.033 19815140

[15] Gabriel DB, Southard KA, Qian F, Marshall SD, Franciscus RG, Southard TE. Cervical vertebrae maturation method: poor reproducibility. Am J Orthod Dentofac Orthop. 2009;136(4):478.e1-7 (**discussion-80**). 10.1016/j.ajodo.2007.08.028 19815136

[16] Predko-Engel A, Kaminek M, Langova K, Kowalski P, Fudalej PS. Reliability of the cervical vertebrae maturation (CVM) method. Bratisl Lek. 2015;116(4):222–6. 10.4149/bll_2015_043 25773948

[17] Shimizu Y, Tanikawa C, Kajiwara T, Nagahara H, Yamashiro T. The validation of orthodontic artificial intelligence systems that perform orthodontic diagnoses and treatment planning. Eur J Orthod. 2022;44(4):436–44. 35050343 10.1093/ejo/cjab083

[18] Jung SK, Kim TW. New approach for the diagnosis of extractions with neural network machine learning. Am J Orthod Dentofac Orthop. 2016;149(1):127–33. 10.1016/j.ajodo.2015.07.030 26718386

[19] Yu JH, Kim JH, Liu J, Mangal U, Ahn HK, Cha JY. Reliability and time-based efficiency of artificial intelligence-based automatic digital model analysis system. Eur J Orthod. 2023;45(6):712–21. 37418746 10.1093/ejo/cjad032

[20] Santiago RC, Cunha AR, Júnior GC, Fernandes N, Campos MJ, Costa LF, et al. New software for cervical vertebral geometry assessment and its relationship to skeletal maturation–a pilot study. Dentomaxillofac Radiol. 2014;43(2):20130238. 24319125 10.1259/dmfr.20130238 PMC4064620

[21] Kök H, Acilar AM, İzgi MS. Usage and comparison of artificial intelligence algorithms for determination of growth and development by cervical vertebrae stages in orthodontics. Prog Orthod. 2019;20(1):41. 31728776 10.1186/s40510-019-0295-8 PMC6856254

[22] Kök H, İzgi MS, Acılar AM. Evaluation of the artificial neural network and Naive Bayes models trained with vertebra ratios for growth and development determination. Turk J Orthod. 2021;34(1):2–9. 33828872 10.5152/TurkJOrthod.2020.20059 PMC7990271

[23] Kök H, Izgi MS, Acilar AM. Determination of growth and development periods in orthodontics with artificial neural network. J Orthod Craniofac Res. 2021;24(S2):76–83. 10.1111/ocr.12443 33232582

[24] Amasya H, Cesur E, Yıldırım D, Orhan K. Validation of cervical vertebral maturation stages: artificial intelligence vs human observer visual analysis. Am J Orthod Dentofac Orthop. 2020;158(6):e173–9. 10.1016/j.ajodo.2020.08.014 33250108

[25] Amasya H, Yildirim D, Aydogan T, Kemaloglu N, Orhan K. Cervical vertebral maturation assessment on lateral cephalometric radiographs using artificial intelligence: comparison of machine learning classifier models. Dentomaxillofac Radiol. 2020;49(5):20190441. 32105499 10.1259/dmfr.20190441 PMC7333473

[26] Makaremi M, Lacaule C, Mohammad-Djafari A. Deep learning and artificial intelligence for the determination of the cervical vertebra maturation degree from lateral radiography. Entropy. 2019;21(12):1222.

[27] Seo H, Hwang J, Jeong T, Shin J. Comparison of deep learning models for cervical vertebral maturation stage classification on lateral cephalometric radiographs. J Clin Med. 2021;10(16):3591. 34441887 10.3390/jcm10163591 PMC8397111

[28] Kim E-G, Oh I-S, So J-E, Kang J, Le VNT, Tak M-K, et al. Estimating cervical vertebral maturation with a lateral cephalogram using the convolutional neural network. J Clin Med. 2021;10(22):5400. 34830682 10.3390/jcm10225400 PMC8620598

[29] Mohammad-Rahimi H, Motamadian SR, Nadimi M, Hassanzadeh-Samani S, Minabi MAS, Mahmoudinia E, et al. Deep learning for the classification of cervical maturation degree and pubertal growth spurts: a pilot study. Korean J Orthod. 2022;52(2):112–22. 35321950 10.4041/kjod.2022.52.2.112 PMC8964471

[30] Atici SF, Ansari R, Allareddy V, Suhaym O, Cetin AE, Elnagar MH. Fully automated determination of the cervical vertebrae maturation stages using deep learning with directional filters. PLoS ONE. 2022;17(7):e0269198. 35776715 10.1371/journal.pone.0269198 PMC9249196

[31] Zhou J, Zhou H, Pu L, Gao Y, Tang Z, Yang Y, et al. Development of an artificial intelligence system for the automatic evaluation of cervical vertebral maturation status. Diagnostics. 2021;11(12):2200. 34943436 10.3390/diagnostics11122200 PMC8700528

[32] Wang S, Huang L, Gao A, Ge J, Zhang T, Feng H, et al. Machine/deep learning for software engineering: a systematic literature review. IEEE Trans Softw Eng. 2023;49(3):1188–231.

[33] LeCun Y, Bengio Y, Hinton G. Deep learning. Nature. 2015;521(7553):436–44. 26017442 10.1038/nature14539

[34] Mathew R, Palatinus S, Padala S, Alshehri A, Awadh W, Bhandi S, et al. Neural networks for classification of cervical vertebrae maturation: a systematic review. Angle Orthod. 2022;92(6):796–804. 36069934 10.2319/031022-210.1 PMC9598845

[35] Erickson BJ, Korfiatis P, Akkus Z, Kline TL. machine learning for medical imaging. Radiographics. 2017;37(2):505–15. 28212054 10.1148/rg.2017160130 PMC5375621

[36] Remeseiro B, Bolon-Canedo V. A review of feature selection methods in medical applications. Comput Biol Med. 2019;112:103375. 31382212 10.1016/j.compbiomed.2019.103375

[37] Priestley M, Od’onnell F, Simperl E. A survey of data quality requirements that matter in ML development pipelines. J Data Inf Qual. 2023;15(2):11.

[38] Ramezan CA, Warner TA, Maxwell AE, Price BS. Effects of training set size on supervised machine-learning land-cover classification of large-area high-resolution remotely sensed data. Remote Sens. 2021;13(3):368.

[39] Ying X. An overview of overfitting and its solutions. J Phys Conf Ser. 2019;1168:022022.

[40] Pedregosa F, Varoquaux G, Gramfort A, Michel V, Thirion B, Grisel O, et al. Scikit-learn: machine learning in Python. J Mach Learn Res. 2011;12:2825–30.

[41] Elgeldawi E, Sayed A, Galal AR, Zaki AM. Hyperparameter tuning for machine learning algorithms used for arabic sentiment analysis. Informatics. 2021;8(4):79.

[42] Brown CE. Coefficient of variation. In: Brown CE, editor. Applied multivariate statistics in geohydrology and related sciences. Berlin, Heidelberg: Springer; 1998. p. 155–7.

[43] McHugh ML. Interrater reliability: the kappa statistic. Biochem Med. 2012;22(3):276–82. PMC3900052 23092060

[44] Santiago RC, de Miranda Costa LF, Vitral RW, Fraga MR, Bolognese AM, Maia LC. Cervical vertebral maturation as a biologic indicator of skeletal maturity. Angle Orthod. 2012;82(6):1123–31. 22417653 10.2319/103111-673.1 PMC8813133

[45] Rainey B-J, Burnside G, Harrison JE. Reliability of cervical vertebral maturation staging. Am J Orthod Dentofac Orthop. 2016;150(1):98–104. 10.1016/j.ajodo.2015.12.013 27364211

[46] Zhao XG, Lin J, Jiang JH, Wang Q, Ng SH. Validity and reliability of a method for assessment of cervical vertebral maturation. Angle Orthod. 2012;82(2):229–34. 21875315 10.2319/051511-333.1 PMC8867953

[47] Schoretsaniti L, Mitsea A, Karayianni K, Sifakakis I. Cervical vertebral maturation method: reproducibility and efficiency of chronological age estimation. Appl Sci. 2021;11(7):3160.

[48] Al-Shayea EI. A survey of orthodontists’ perspectives on the timing of treatment: a pilot study. J Orthod Sci. 2014;3(4):118–24. 25426455 10.4103/2278-0203.143232 PMC4238079

[49] Makrygiannakis MA, Giannakopoulos K, Kaklamanos EG. Evidence-based potential of generative artificial intelligence large language models in orthodontics: a comparative study of ChatGPT, Google Bard, and Microsoft Bing. Eur J Orthod. 2024. 10.1093/ejo/cjae017 PMC12810200 38613510

[50] Montasser MA, Viana G, Evans CA. Racial and sex differences in timing of the cervical vertebrae maturation stages. Am J Orthod Dentofac Orthop. 2017;151(4):744–9. 10.1016/j.ajodo.2016.09.019 28364898

