# Correction to: Classification of cervical vertebral maturation stages with machine learning models: leveraging datasets with high inter- and intra-observer agreement

**DOI:** 10.1186/s40510-025-00567-1

**Published:** 2025-06-11

**Authors:** Potjanee Kanchanapiboon, Pitipat Tunksook, Prinya Tunksook, Panrasee Ritthipravat, Supatchai Boonpratham, Yodhathai Satravaha, Chaiyapol Chaweewannakorn, Supakit Peanchitlertkajorn

**Affiliations:** 1 Division of Nuclear Medicine, Department of Radiology, Faculty of Medicine Siriraj Hospital, Mahidol University, 2 Wang Lang Rd, Siriraj, Bangkok Noi, Bangkok, 10700 Thailand; 2 Department of Orthodontics, Faculty of Dentistry, Mahidol University, 6 Yothi Rd, Thung Phaya Thai, Ratchathewi, Bangkok, 10400 Thailand; 3 Private Practice, Bangkok, Thailand; 4 Department of Biomedical Engineering, Faculty of Engineering, Mahidol University, 999 Phutthamonthon 4 Rd, Salaya, Nakhon Pathom 73170 Thailand

**Correction to: Progress in Orthodontics 25: 35 (2024)**

10.1186/s40510-024-00535-1

Following publication of the original article [1], the authors identified that the IRB approval number was incorrectly indicated in the Methods and the Ethics approval and consent to participate section.

The Methods section currently reads:

**Methods**

- The study protocol was approved by the Mahidol University Institutional Review Board, Faculty of Dentistry/Pharmacy, with the approval number MU-DT/PY-IRB 2022/0.15.2803.

The Methods section should read:

**Methods**

- The study protocol was approved by the Mahidol University Institutional Review Board, Faculty of Dentistry/Pharmacy, with the approval number CoA No. MU-DT/PY-IRB 2022/015.2803.

The section currently reads:

**Ethics approval and consent to participate**

- The study protocol was approved by the Mahidol University Institutional. Review Board, Faculty of Dentistry/Pharmacy, with the approval number. MU-DT/PY-IRB 2022/0.15.2803.

The section should read:

**Ethics approval and consent to participate**

- The study protocol was approved by the Mahidol University Institutional. Review Board, Faculty of Dentistry/Pharmacy, with the approval number. CoA No. MU-DT/PY-IRB 2022/015.2803.

The Methods and Ethics approval and consent to participate have been updated above and the original article [1] has been corrected.
